# California State Veterinary Medical Association

**Published:** 1892-04

**Authors:** R. A. Archibald

**Affiliations:** Secretary; Sacramento, Cal.


### SOCIETY PROCEEDINGS.

*California State Veterinary Medical Association.*—The quarterly meeting of the California State Veterinary Medical Association was held at the Baldwin Hotel, San Francisco, on Wednesday evening, March 9th, 1892.

Vice-President Dr. W. F. Egan presided in the absence of President Dr. W. E. D. Morrison, of Los Angeles. Upon roll call the following gentlemen answered to their names, viz.: Drs. McCollum, Pierce, Orvis, H. A. Spencer, H. F. Spencer, Fox, Wadams, Davenport, Burns, Egan and Maclay. The minutes of the previous meeting were read and approved. Letters of regret from absent members were then read. A letter was received from Mrs. W. H. Woodruff, thanking the Association for sympathy and financial help rendered to her on the death of her late husband. There being no papers to be read, a general discussion on subjects of interest to the profession took place. The subject matter of "Glanders" was taken up and considered by the members Dr. Maclay again urged upon the members the necessity of having proper sanitary laws passed. Drs. Fox, Burns, McCollum, Orvis, Pierce, Spencer and Egan all testified to the need of proper legislative power to stamp out the many and virulent diseases of horses, cattle and swine, and testified to the spread of these diseases in the countries of San Joaquin, Santa Barbara, Sacramento, Sonoma, Santa Cruz and Mendocino. It was stated that in those counties where the supervisors had passed ordinances and appointed county veterinarians the disease had been nearly rooted out, and was far less prevalent than in counties where no action had been taken by the authorities.

The subject of "Influenza" was taken up and considered. Dr. Maclay spoke at some length on the disease, and other members followed.

The Vice-President stated that Dr. McNult, a prominent physician of San Francisco, had been, and is now, agitating the starting of a veterinary college in that city. This met with the hearty approval of the members. There being no further business before the meeting, on motion the meeting adjourned to meet on June 8th, 1892.

R. A. Archibald, D. V. S., *Secretary.*

Sacramento, Cal.

